# Surgical Site Infections and Antimicrobial Resistance: Six Years of Data from a Western Romanian Hospital

**DOI:** 10.3390/medicina62010108

**Published:** 2026-01-03

**Authors:** Catalin Vladut Ionut Feier, Ana Teodor, Calin Muntean, Oliana Cristina Faităr, Corina Iuliana Cilibiu, Narcisa Jianu, Delia Muntean, Valentina Buda, Vasile Gaborean, Marius Murariu

**Affiliations:** 1 Abdominal Surgery and Phlebology Research Center, “Victor Babeş” University of Medicine and Pharmacy, 300041 Timişoara, Romania; catalin.feier@umft.ro (C.V.I.F.); murariu.marius@umft.ro (M.M.); 2 First Surgery Clinic, “Pius Brinzeu” Clinical Emergency Hospital, 300723 Timişoara, Romania; 3 Doctoral School, “Victor Babeş” University of Medicine and Pharmacy, 300041 Timişoara, Romania; narcisa.dinu@umft.ro; 4 Multidisciplinary Research Center of Antimicrobial Resistance, Department of Microbiology, Faculty of Medicine, “Victor Babeş” University of Medicine and Pharmacy, 300041 Timişoara, Romania; muntean.delia@umft.ro; 5 Medical Informatics and Biostatistics, Department III-Functional Sciences, “Victor Babeş” University of Medicine and Pharmacy, 300041 Timişoara, Romania; cmuntean@umft.ro; 6 Faculty of Medicine, “Victor Babeş” University of Medicine and Pharmacy, 300041 Timişoara, Romania; oliana.faitar@student.umft.ro (O.C.F.); cilibiu.corina14@gmail.com (C.I.C.); 7 Faculty of Pharmacy, “Victor Babeş” University of Medicine and Pharmacy, 300041 Timişoara, Romania; 8 Research Center for Pharmaco-Toxicological Evaluation, “Victor Babeş” University of Medicine and Pharmacy, 300041 Timişoara, Romania; 9 Institute of Cardiovascular Diseases Timisoara, 300310 Timişoara, Romania; 10 Thoracic Surgery Research Center, “Victor Babeş” University of Medicine and Pharmacy, 300041 Timişoara, Romania; vasile.gaborean@umft.ro; 11 Department of Surgical Semiology, Faculty of Medicine, “Victor Babeş” University of Medicine and Pharmacy, 300041 Timişoara, Romania

**Keywords:** open surgery, COVID-19 pandemic, *Pseudomonas aeruginosa*, Enterococcus spp., fluoroquinolones, antimicrobial stewardship

## Abstract

*Background and Objectives*: The onset of the COVID-19 pandemic posed a new challenge to hospital infection prevention measures and to the antimicrobial therapies adopted. The present study aimed to assess the influence of the COVID-19 pandemic on the dynamics of surgical site infection (SSI) rates and the variations in the microbiological profiles of the SSI. *Materials and Methods*: A retrospective, single-center study was conducted to examine data from patients who underwent conventional surgical procedures and developed SSI. The study was conducted at the First Surgery Clinic of the “Pius Brinzeu” Clinical Emergency Hospital, Timisoara, Romania. Data from 173 patients were analyzed over six years (from 26 February 2018 to 25 February 2024). The selected time interval was divided into three periods: pre-pandemic, pandemic, and post-pandemic. *Results*: During the pandemic, the average patient age was significantly lower than in the other periods. The average length of stay decreased consistently over the six-year study period. Among the 173 patients included in the study, 71.1% had a monobacterial infection, while the remaining 28.9% had infections involving at least two different bacteria. The two most commonly identified bacteria in more than 50% of the cases were *Pseudomonas aeruginosa* and *Enterococcus* spp. There was a significant decrease in bacterial resistance to levofloxacin and ciprofloxacin over the study period, with resistance dropping from 50% (pre-pandemic) and 53.3% (pandemic) to just 9.1% (post-pandemic). *Conclusions*: The COVID-19 pandemic substantially altered the SSI profile in our institution. The temporary increase in SSI frequency during the pandemic was likely related to shifts in surgical case mix and care delivery, rather than decreased infection control performance. Post-pandemic restoration of surgical flow coincided with improved antimicrobial susceptibility patterns, particularly for fluoroquinolones. Microbiological surveillance, the use of infection prevention measures, and robust stewardship initiatives remain essential to maintain these favorable trends and mitigate the emergence of future resistance.

## 1. Introduction

Surgical Site Infections are among the most common types of Hospital-Acquired Infections (HAI) [1,2,3]. SSIs are known to have not only immediate but also long-term consequences. They often lead to life-threatening complications, prolongation of hospitalization, and an increase in recurrence rate and low survival rates, adding to the overall treatment costs [2,3,4,5].

Along with physical impairment resulting from SSI as a surgical complication, there is also a significant impact on patients’ mental well-being, resulting in anxiety and depression, and ultimately affecting their quality of life [4,6].

The Centers for Disease Control and Prevention (CDC) definition of SSI is the most widely accepted, dividing SSIs into three groups: superficial, deep, and organ site infections [7].

Superficial SSIs occur within 30 days following the surgical procedure. They involve only the skin or subcutaneous tissue at the site of the incision and must meet at least one of the following criteria: purulent drainage from the surgical incision; a positive microbiological culture; or the presence of one or more signs and symptoms of infection, such as tenderness, swelling, heat, or redness [7,8].

Deep SSIs can develop within 30 to 90 days after surgery and involve the fascial and muscular layers. For a deep SSI to be identified, the patient must exhibit one or more of the following: purulent drainage from the deep incision; a deep incision that either spontaneously dehisces or is deliberately opened by a physician, accompanied by a positive microbiological culture from the deep soft tissue; or evidence of infection involving the deep surgical incision [7].

Organ/Space SSI can occur within the first 30 to 90 days after surgery. It affects the tissues of the organ or space manipulated during the procedure and is related to the surgery. A patient may have one or more of the following signs indicating an infection or abscess involving the organ or space tissue: evidence of infection detected through a physical examination, imaging tests, or histopathological analysis; purulent drainage from the organ or space tissue drain; a positive microbiological culture from fluid or tissue in the affected organ or space [7,8].

Different countries have varying rates of SSIs, which are primarily influenced by their level of economic development and overall healthcare conditions. Generally, high-income countries report lower SSI rates compared to low- and middle-income countries. Among all types of surgeries, abdominal surgery has the highest incidence of SSI. Moreover, elective abdominal surgeries tend to have a lower SSI rate than emergency abdominal surgeries [9].

Beyond incidence rates, the clinical burden of SSIs is increasingly shaped by the global rise in antimicrobial resistance (AMR), which complicates therapeutic management and worsens patient outcomes. Regional AMR data from the World Health Organization (WHO) European Region burden analysis (2019) estimated approximately 541,000 deaths associated with bacterial AMR, with the highest resistance burden observed in Eastern and Southern Europe, including Romania. Particularly concerning resistance patterns were reported for carbapenem-resistant *Enterobacterales* and *Pseudomonas aeruginosa* [10]. Several studies conducted in western regions of Romania have identified a higher incidence of infections caused by *Staphylococcus aureus*, *Escherichia coli*, and *Klebsiella* spp., alongside a lower incidence of *Pseudomonas aeruginosa* and *Enterococcus* spp., albeit with heterogeneous antimicrobial resistance profiles [11,12,13,14]. Isolated *Pseudomonas aeruginosa* strains were predominantly resistant to cephalosporins, carbapenems, and fluoroquinolones [14,15], while *Enterococcus* spp. commonly exhibited resistance to cefuroxime, fluoroquinolones, and macrolides but remained susceptible to glycopeptides and linezolid [13]. Notably, certain carbapenem-resistant *Pseudomonas aeruginosa* strains demonstrated an enhanced capacity for biofilm formation, emphasizing the importance of accurate strain identification and characterization [16].

An often-overlooked contributor to the AMR burden is represented by water-borne microorganisms, which are frequently multidrug-resistant (MDR). Most *P. aeruginosa* strains isolated from hospital wastewater systems have been identified as carriers of metallo-β-lactamases (MBLs) and extended-spectrum β-lactamases (ESBLs) [17,18]. Additionally, the long-term persistence of MDR organisms on hospital surfaces represents a continuous infection control challenge, with pathogens such as *Acinetobacter baumannii*, *Pseudomonas aeruginosa*, *Cutibacterium acnes*, and *Staphylococcus epidermidis* frequently detected across various hospital environments [19].

In this context of rising MDR pathogen prevalence, well-implemented and adequately funded AMR action plans are essential. Addressing limitations in microbiological diagnostic capacity, improving AMR awareness, and strengthening antimicrobial stewardship programs can significantly reduce mortality attributable to antimicrobial resistance [10,20].

Antimicrobial stewardship programs have already demonstrated a substantial impact on reducing the development of multidrug-resistant bacteria and on overall costs of patients’ antiinfective treatment, reducing drug costs, length of stay, and readmission rates [21,22]. The onset of the COVID-19 pandemic posed a new challenge to infection prevention measures within hospitals and to the antimicrobial therapies adopted, significantly influencing antimicrobial stewardship programs [23,24].

The COVID-19 pandemic significantly impacted healthcare services worldwide. To safeguard patients and healthcare personnel and prevent the spread of SARS-CoV-2, many surgical procedures had to be postponed. In Romania, authorities implemented recommendations from the World Health Organization (WHO), adopting measures similar to those seen in other countries affected by COVID-19 to curb the virus’s rapid transmission [25].

It is estimated that approximately 28.4 million elective surgical procedures were globally canceled during this time period [26,27]. These delays presented both advantages and disadvantages. On the positive side, postponing surgical interventions helped reduce the risk of patients contracting a SARS-CoV-2 infection during the perioperative period, which could have put about half of these patients at risk for developing pulmonary complications after surgery [26]. However, for specific patient groups, such as those with cancer, venous ulcers, or soft-tissue infections, delaying treatment could significantly worsen their conditions and potentially lead to life-threatening complications, considerably reducing the survival rate [28,29,30,31,32].

Conventional open surgical procedures carry the highest risk of infectious complications [8,33]. This six-year retrospective study aims to evaluate the trends in surgical site infections following conventional surgical interventions across three distinct timeframes, including the COVID-19 pandemic. A key objective of the study is to identify shifts in the microbiological profiles and antimicrobial resistance patterns of SSI-associated pathogens, highlighting the importance of ongoing antimicrobial surveillance to optimize postoperative outcomes.

Although previous studies have examined the impact of the COVID-19 pandemic on surgical activity and SSIs, data specifically addressing conventional surgeries and antimicrobial resistance trends before, during, and after the pandemic remain limited. Furthermore, there is a paucity of regional data regarding antimicrobial surveillance of SSIs in Western Romania. By analyzing resistance patterns over six years, this study seeks to provide valuable insights into the local AMR landscape, to support infection control strategies, guide antimicrobial therapy, and ultimately improve patient safety.

## 2. Materials and Methods

### 2.1. Study Design and Setting

This is a retrospective, single-center, cross-sectional observational study conducted in the First Surgery Clinic, a General Surgery Unit within the Emergency County Hospital of Timișoara, Romania. The hospital is a tertiary referral center providing emergency and elective general surgical care for the city of Timișoara and the surrounding region.

The study analyzed data from inpatients who developed SSIs following conventional surgical procedures performed at the First Surgery Clinic of the “Pius Brînzeu” Clinical Emergency Hospital, Timișoara, Romania. A total of 173 consecutive patients hospitalized between 26 February 2018 and 25 February 2024 met the inclusion criteria and were included in the analysis. Data extraction and validation were performed over five months (October 2024–February 2025).

The date 26 February 2020 was selected as the index point marking the first confirmed COVID-19 case in Romania, while 8 March 2022 represented the lifting of all pandemic-related restrictions. Based on these epidemiological milestones, patients were stratified into three consecutive observational cohorts for comparative analysis of SSI incidence, clinical outcomes, microbiological characteristics, and antimicrobial resistance profiles:

- Pre-pandemic period: 26 February 2018–25 February 2020;
- Pandemic period: 26 February 2020–25 February 2022;
- Post-pandemic period: 26 February 2022–25 February 2024.

### 2.2. Eligibility Criteria and Participant Selection

Patients were eligible for inclusion if they met all of the following criteria:

1. Underwent conventional surgical procedures in the clinic during the study period and subsequently developed an SSI.
2. Had available microbiological cultures from the surgical wound and corresponding antibiotic susceptibility data (antibiograms).
3. Tested negative for SARS-CoV-2 both at admission and throughout hospitalization.

Patients were excluded if they lacked complete microbiological or clinical data, had undergone reoperation during the same admission, or were transferred from another facility after surgery.

All eligible consecutive patients meeting the predefined criteria during the study period were included. No sampling, matching, or randomization procedures were applied.

SSIs were defined according to the Centers for Disease Control and Prevention (CDC) criteria, encompassing superficial, deep incisional, and organ/space infections [7].

### 2.3. Patient Screening and Hospital Protocol

During the pandemic, all patients scheduled for elective surgery underwent SARS-CoV-2 Reverse Transcription Polymerase Chain Reaction (RT-PCR) testing using nasopharyngeal swabs in accordance with national and institutional guidelines. Negative RT-PCR results obtained externally within 24 h before admission were accepted, though infrequently used due to cost considerations. Rapid antigen tests were not accepted.

Patients awaiting RT-PCR results were isolated in dedicated rooms, temporarily reducing hospital capacity, as four rooms were reserved exclusively for pre-admission isolation. Patients testing negative were transferred to standard wards for preoperative preparation. In contrast, positive cases were redirected to designated COVID-19 treatment units (moderate/severe cases) or instructed to isolate at home for 14 days (mild/asymptomatic cases).

Following the lifting of restrictions on 8 March 2022, the clinic progressively resumed regular activity, with standard admission and hospitalization protocols reinstated.

### 2.4. Ethical Approval, Data Collection, and Management

Ethical approval was obtained from the Hospital Ethics Committee (548/23 June 2025), and the investigation adhered to the principles of the Declaration of Helsinki.

Patient data were retrieved from the institutional Electronic Medical Record (EMR) system. Three independent researchers manually extracted data, and all variables were entered into a standardized Microsoft Excel^®^ database (Microsoft Corp., Redmond, WA, USA). To ensure data integrity, two independent researchers performed double data entry, followed by a random cross-check by a third investigator. Discrepancies were resolved by consensus before final database consolidation.

Only patients meeting the predefined inclusion criteria were analyzed. All data were anonymized before analysis and handled in compliance with the European Union General Data Protection Regulation (EU GDPR).

Moreover, the reporting of this study is in accordance with the Strengthening the Reporting of Observational Studies in Epidemiology (STROBE) checklist for cross-sectional studies [34].

### 2.5. Variables Collected

The dataset included the following categories:

- Demographic variables: age, sex, and residence (urban/rural).
- Clinical data: length of hospitalization, interval between admission and wound sampling, surgery type (emergency vs. elective), postoperative ICU admission, and in-hospital mortality.
- Primary diagnosis: categorized into seven groups—abdominopelvic neoplasms, acute abdomen, abdominal wall defects, chronic limb-threatening ischemia, soft-tissue infections, venous leg ulcers, and other conditions.
- Comorbidities: assessed using the Charlson Comorbidity Index (CCI) [35,36].
- Microbiological findings: pathogen identification, monomicrobial vs. polymicrobial infection status, and antibiotic susceptibility profiles.

The primary outcome of the study was the distribution of surgical site infection–associated pathogens and their antimicrobial resistance patterns across the three predefined study periods (pre-pandemic, pandemic, and post-pandemic). Secondary outcomes included temporal changes in the frequency of surgical site infections over the study period; patient demographic and clinical characteristics, such as age, sex, comorbidity burden, and primary admission diagnosis; hospitalization-related outcomes, including length of hospital stay, postoperative intensive care unit admission, and in-hospital mortality; as well as the distribution of SSI-associated pathogens according to diagnostic category and type of surgical intervention (emergency versus elective).

### 2.6. Microbiological Analysis

Bacterial isolates were identified by the hospital’s microbiology laboratory using standard diagnostic methods, and antibiotic susceptibility was interpreted according to the Clinical and Laboratory Standards Institute (CLSI) guidelines. Microbiological identification techniques and susceptibility testing protocols remained unchanged throughout the study period, ensuring consistency and comparability across all cohorts.

### 2.7. Statistical Analysis

All statistical analyses were performed using IBM SPSS Statistics version 25.0 (IBM Corp., Armonk, NY, USA). A significance level of *p* < 0.05 was adopted. Continuous variables were expressed as mean ± standard deviation (SD), while categorical variables were summarized as absolute values and percentages (%).

**Figure 1:**
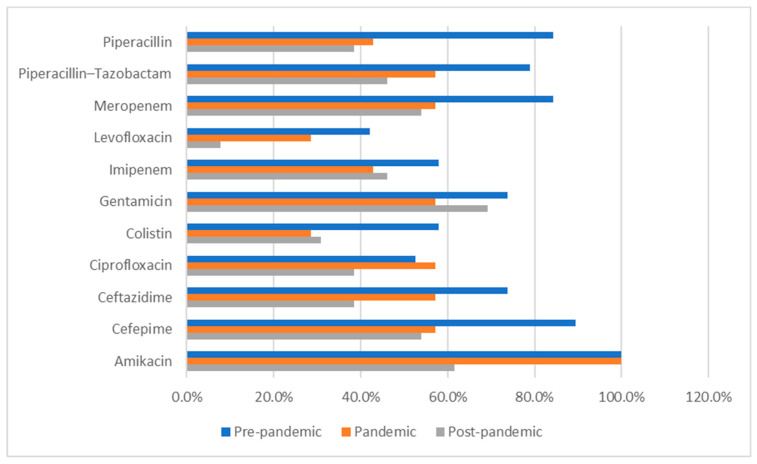
*Pseudomonas aeruginosa* antibiotic resistance profile across the studied periods. Percentages represent antimicrobial susceptibility rates and refer to the proportion of tested isolates that were susceptible to each antibiotic during each study period.

Prior to analysis, the distribution of continuous data was assessed using the Shapiro–Wilk test to determine normality. Based on the outcome, parametric or non-parametric tests were applied following a structured decision process, as outlined in Figure 1 of Sayed AA [37]. For normally distributed data, comparisons between two groups were performed with Student’s *t*-test, while comparisons involving more than two groups used one-way ANOVA, with Levene’s test for variance homogeneity and Bonferroni post hoc correction where appropriate. For abnormally distributed data, the Mann–Whitney U test or Kruskal–Wallis test was applied.

Categorical variables were analyzed using the Chi-square test, or Fisher’s exact test when expected cell counts were <5. Continuous variables were not arbitrarily categorized unless clinically justified. Missing data were managed through listwise deletion, without data imputation. All results were presented with 95% confidence intervals (CI). No sensitivity or subgroup analyses were performed.

## 3. Results

After applying the inclusion criteria, 173 patients who developed SSIs following conventional surgical interventions were included in the analysis.

### 3.1. Demographic and Clinical Characteristics

Across the six-year study period, the case distribution was as follows (Table 1): pre-pandemic: 72 patients (41.6%); pandemic: 48 patients (27.7%); post-pandemic: 53 patients (30.6%).

The mean age of the study population was 64.6 ± 12.4 years, with no statistically significant variation across the three periods (*p* = 0.117). However, pairwise comparison revealed that patients in the pandemic cohort were significantly younger than those in the pre-pandemic period (62.29 ± 14.72 vs. 66.92 ± 10.36 years, *p* = 0.046).

Gender distribution showed no significant differences (*p* = 0.149), with males representing 43.1%, 60.4%, and 45.3% of cases in the pre-, pandemic, and post-pandemic periods, respectively.

Similarly, the urban-to-rural ratio remained stable (*p* = 0.907), as did the proportion of patients with Charlson Comorbidity Index > 3 (51.4%, 39.6%, and 41.5%; *p* = 0.400).

### 3.2. Hospitalization Parameters

The mean length of hospital stay was 45.16 ± 26.08 days, showing a statistically significant reduction across the three periods (*p* = 0.003): pre-pandemic: 52.26 ± 28.85 days; pandemic: 44.27 ± 25.63 days; post-pandemic: 36.3 ± 19.21 days.

The time interval between admission and wound sampling did not differ significantly (*p* = 0.713): pre-pandemic: 16.55 ± 10.78 days; pandemic: 16.75 ± 11.39 days; post-pandemic: 15.18 ± 9.24 days.

### 3.3. Diagnostic Categories

Admission diagnosis of the patients is presented in Table 2.

Primary admission diagnoses (presented in Table 2) varied significantly among the three study periods (*p* = 0.012) as follows:

- pre-pandemic: chronic limb-threatening ischemia (36 patients, 50.0%), abdominopelvic neoplasms (14, 19.4%), and abdominal wall defects (8, 11.1%);
- pandemic: chronic limb-threatening ischemia (14, 29.2%), soft-tissue infections (9, 18.8%), and venous leg ulcers (6, 12.5%);
- post-pandemic: chronic limb-threatening ischemia (15, 28.3%), soft-tissue infections (11, 20.8%), and abdominopelvic neoplasms (10, 18.9%).

### 3.4. Type of Surgical Intervention

Out of all patients, 99 (57.2%) required emergency surgical procedures, while 74 (42.8%) underwent elective surgery.

The distribution differed significantly across periods (*p* = 0.001): pre-pandemic: 37 patients (51.4%); pandemic: 21 patients (43.8%); post-pandemic: 41 patients (77.4%).

### 3.5. Microbiological Findings

Of the 173 SSI cases, 123 (71.1%) had monobacterial cultures, and 50 (28.9%) had polymicrobial cultures.

The proportion of monobacterial infections was comparable across the three cohorts (*p* = 0.737) and did not vary significantly: pre-pandemic: 70.8% (51/72), pandemic: 75.0% (36/48), post-pandemic: 67.9% (36/53).

Table 3 presents the distribution of bacterial species identified across the three periods, regardless of whether infections were mono- or polymicrobial, with percentages reflecting the relative frequency of each bacterial isolate.

The most frequently isolated pathogens throughout the study were *Pseudomonas aeruginosa* and *Enterococcus* spp. (*Enterococcus faecium* and *Enterococcus faecalis*), together accounting for approximately 45% of all isolates. Other common isolates included *Escherichia coli*, *Staphylococcus aureus*, *Klebsiella* spp., and *Proteus mirabilis* (Table 3).

Bacteria identified with a frequency of less than 2 were not included in the statistical analysis. These bacteria are: Coagulase-negative staphylococci, *Corynebacterium striatum*, *Enterobacter cloacae*, *Streptococcus constellatus*, *Streptococcus agalactiae*, *Proteus vulgaris*, *Candida albicans*, *Citrobacter freundii*, *Bacillus cereus*, *Streptococcus anginosus*, *Providencia stuartii*, *Serratia marcescens*, *Proteus hauseri*, and *Providencia rettgeri*.

### 3.6. Pathogen Distribution by Diagnostic Category

Across the entire six-year study period, *Pseudomonas aeruginosa* and *Enterococcus* spp. (*Enterococcus faecium* and *Enterococcus faecalis*) were the most frequently isolated pathogens, together accounting for approximately 45% of all surgical site infections.

Given their high prevalence and clinical relevance, these two bacterial species were selected for a separate comparative analysis of diagnostic distribution across the pre-pandemic, pandemic, and post-pandemic periods.

Table 4 summarizes the findings in *Pseudomonas aeruginosa* incidence in all 7 diagnoses, across the three studied periods.

Table 5 summarizes the findings in *Enterococcus* spp. incidence across the three studied periods.

### 3.7. Antibiotic Resistance Profiles

**Figure 2:**
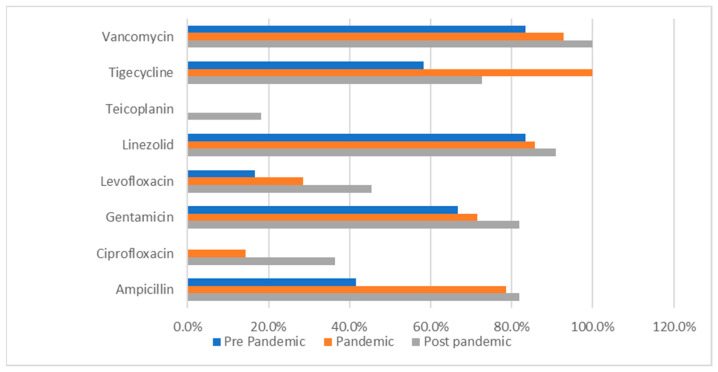
*Enterococcus* spp. antibiotic resistance profile across the studied periods. Percentages represent the proportion of *Enterococcus* spp. isolates resistant to each antimicrobial agent among the isolates tested in each study period.

The following data (presented in Table 6, Table 7, Table 8, Table 9, Table 10 and Table 11) summarizes the antibiotic resistance profile for the most frequently encountered bacterial species identified over the study period, *Pseudomonas aeruginosa* (39 positive cultures) and *Enterococcus* spp. (38 positive cultures). Their antibiotic susceptibility is presented in Figure 1 (for *Pseudomonas aeruginosa*) and Figure 2 (for *Enterococcus* spp.).

Susceptibility to key antibiotics decreased gradually over time, as depicted in Table 6. Although not statistically significant (*p* > 0.05), clinically relevant changes were noted as follows:

- Amikacin: 100% (pre- and pandemic) → 61.5% (post-pandemic);
- Cefepime: 89.5% → 57.1% → 53.8%;
- Ceftazidime: 73.7% → 57.1% → 38.5%;
- Piperacillin/Tazobactam: 78.9% → 57.1% → 46.2%.

A slight decline in carbapenem susceptibility (imipenem, meropenem) was also observed (Table 6).

Intermediate resistance was most frequent for ceftazidime, piperacillin/tazobactam, and ciprofloxacin during the post-pandemic period (Table 6).

*Enterococcus* spp. isolates maintained high resistance rates to linezolid (83.3% → 90.9%) and vancomycin (83.3% → 100%), as presented in Table 7 and Figure 2.

Table 10 presents a comparative analysis of the bacterial resistance profile across the 3 timeframes for *Enterococcus* spp.. It revealed statistically significant differences in the resistance profiles to 2 fluoroquinolones. For ciprofloxacin, the resistance rate was 50.0% in the pre-pandemic period, 53.3% in the pandemic period, and 9.1% in the post–pandemic period (*p* = 0.030). The same trend was observed for levofloxacin, with the resistance rate decreasing from 50.0% and 53.3% in the first two timeframes to 9.1% in the post-pandemic period (*p* = 0.030). These results indicate a notable decline in bacterial resistance to fluoroquinolones following the pandemic.

Resistance to beta-lactams (amoxicillin/clavulanic acid, ampicillin) fluctuated without statistical significance.

The antibiotic intermediate sensitivity is presented in Table 11.

### 3.8. Clinical Outcomes

After analyzing the collected data, five cases (2.89%) required postoperative ICU hospitalization. In the pre-pandemic period, four patients (5.6%) required ICU postoperative monitoring; during the pandemic, only one patient (2.1%) was moved to the ICU; and post-pandemic, none of the post-surgical patients required monitoring. The results show a significant difference across the three studied periods, but they must be interpreted cautiously due to low representation (frequency < 5).

During the study, five patients died in the postoperative period. In the first period, no deaths were recorded. During the pandemic, three patients (6.3%) died, and after the COVID period, two deaths (3.8%) were recorded. The results show a significant difference between the three studied periods (*p* = 0.034), but these results must be interpreted cautiously due to the low representation (frequency < 5).

## 4. Discussion

This six-year retrospective study investigated the impact of the COVID-19 pandemic on the incidence, microbiological profiles, and antimicrobial resistance patterns of SSIs among patients undergoing conventional surgical procedures at a tertiary center in Romania. To eliminate potential confounding factors related to SARS-CoV-2-induced immunosuppression and an increased risk of postoperative complications, patients with a history of or current COVID-19 were excluded from the study [38,39,40,41].

The incidence of SSIs increased significantly during the pandemic and later declined. This rise was likely due to a shift in the types of cases being treated rather than a decrease in aseptic standards. Between 2020 and 2022, elective surgeries were suspended, leading to a predominance of emergency cases such as perforated appendicitis and chronic limb-threatening ischemia, which are associated with a higher risk of SSIs [42]. Similar reports suggest that the increased SSI rates during the pandemic were linked to delayed presentations and a greater number of urgent or contaminated procedures [43,44]. After elective surgeries resumed in March 2022, both the number of SSIs and the length of hospital stays decreased, indicating improved perioperative organization and a return to established infection-prevention protocols.

The age of patients also decreased during the pandemic, likely because older adults avoided hospital care due to fears of SARS-CoV-2 exposure, a trend reported in other studies [45]. The average length of hospital stay decreased after the pandemic, from 52.3 days to 36.3 days. This change aligns with improved perioperative management and more stringent recovery protocols in hospitals. Similar international data indicate that hospital stays were shortened during this time, particularly with the implementation or enhancement of Enhanced Recovery After Surgery (ERAS) measures [46,47,48]. Throughout all three periods studied, *Pseudomonas aeruginosa* and *Enterococcus* spp. were the most common pathogens, collectively accounting for nearly half of the isolates. The high incidence of *P. aeruginosa* is associated with a preference for moist, ischemic tissues, which are often found in cases of limb-threatening ischemia and chronic ulcers [49,50]. Although some studies indicated lower rates of *P. aeruginosa* infections during the pandemic [51,52], our findings demonstrate that this pathogen persisted. This underscores the necessity for targeted surveillance in high-risk surgical units.

Recent multicenter surveillance studies and systematic reviews have reported heterogeneous trends regarding the incidence and antimicrobial resistance of *Pseudomonas aeruginosa* during the COVID-19 pandemic. While some institutions described a transient decline in multidrug-resistant *P. aeruginosa* during interwave periods [51,53], others reported increased resistance to carbapenems and fluoroquinolones, particularly in the post-pandemic phase [52,54]. Extensive ecological analyses and meta-analyses further suggest that pandemic-related disruptions amplified regional variability in *P. aeruginosa* epidemiology rather than producing uniform global trends, with resistance increasing predominantly in settings where antimicrobial stewardship and infection control programs were compromised [55,56].

In contrast to studies reporting a pandemic-associated decline, our data show a persistent presence of *P. aeruginosa* across all three periods, with no statistically significant reduction during the pandemic. This finding is consistent with reports focusing on high-risk surgical populations and SSI cohorts [57,58] and may be explained by the specific case mix of our cohort, which included a high proportion of patients with chronic limb-threatening ischemia, venous leg ulcers, and soft-tissue infections. These conditions provide a favorable microenvironment for *P. aeruginosa* colonization and biofilm formation and may outweigh the potential impact of intensified infection control measures implemented during the pandemic [43,49].

Taken together, these observations indicate that pandemic-era trends in *P. aeruginosa* incidence and resistance are highly context-dependent and influenced by underlying patient pathology, environmental persistence, and continuity of antimicrobial stewardship. Our findings, therefore, complement existing literature by highlighting that reductions in *P. aeruginosa* incidence reported during the pandemic may not be generalizable to surgical units managing predominantly ischemic or chronic wound-related conditions [59,60].

A relative increase in *Enterococcus* spp. was observed during the pandemic, consistent with the organism’s environmental resilience and tolerance to disinfectants heavily used during this period [55,61,62,63,64]. Post-pandemic patterns suggest a partial return toward baseline microbiological ecology. More specifically, several studies have reported an increased prevalence of *Enterococcus* spp. during the COVID-19 pandemic, frequently linked to intensified disinfectant use, prolonged hospitalization, and increased exposure to broad-spectrum antibiotics [55,61,62,63,64]. Conversely, other reports did not observe significant changes in *Enterococcus* incidence but described marked shifts in antimicrobial resistance patterns, particularly toward glycopeptides and oxazolidinones [55,63,64].

A significant finding is the marked decline in fluoroquinolone resistance among *Enterococcus* spp. following the pandemic, with *p*-values of 0.030 for both ciprofloxacin and levofloxacin. This contrasts with global reports of increasing resistance, often linked to antibiotic overuse in COVID-19 wards [26,65]. Contributing factors to this decline may include improved antimicrobial stewardship and a decrease in the empirical use of fluoroquinolones after the pandemic. However, resistance to vancomycin and linezolid remains high, exceeding 80%, which is consistent with global trends [55].

*P. aeruginosa* showed a gradual loss of susceptibility to β-lactams (ceftazidime, piperacillin-tazobactam, carbapenems), consistent with international surveillance [57], although differences were not statistically significant. The rise in imipenem resistance reported in other settings underscores the species’ adaptability and the need for continuous local monitoring [51,52,54].

Only 2.9% of patients required postoperative intensive care, and mortality remained low, though small numbers limit definitive conclusions. Notably, extensive international collaborative studies have demonstrated that postoperative mortality was significantly higher during the COVID-19 pandemic compared to the pre-pandemic period. The STARSurg and COVIDSurg Collaborative study showed that the adjusted odds of death following surgery were higher during the pandemic, primarily driven by pulmonary complications, suggesting that SARS-CoV-2 infection could have acted as a major confounding factor for postoperative outcomes, including surgical site infections [66]. This evidence supports our methodological decision to exclude patients with active or prior SARS-CoV-2 infection to minimize confounding and enable a more accurate assessment of SSI-related epidemiology and antimicrobial resistance patterns.

The study’s retrospective, single-center design limits its generalizability. Additionally, the lack of data on operative volumes prevented normalization of the SSI incidence. Reliance on electronic medical records introduces the potential for information bias. Furthermore, wound classification and prophylactic strategies were not analyzed, and the causal link between the COVID-19 pandemic and the observed changes cannot be established. Another limitation is that the predefined study periods may not perfectly reflect real-world surgical activity, as the impact of the COVID-19 pandemic on surgical scheduling and hospital workflows may not have aligned precisely with the selected timeframes. Finally, only patients diagnosed with SSIs were included in the analysis, which precluded calculation of SSI subtype rates relative to the total surgical population. Future prospective studies including all surgical patients, regardless of infection status, would allow a more detailed evaluation of temporal trends in SSI subtypes. Despite these limitations, the study provides valuable insights into the epidemiology of SSIs and trends in resistance across the pre-pandemic, pandemic, and post-pandemic periods.

The post-pandemic increase in fluoroquinolone susceptibility highlights the importance of structured antimicrobial stewardship. To maintain these improvements, continuous surveillance and adaptive stewardship strategies are necessary. Reinforcing CDC-aligned surgical site infection prevention bundles—such as timely prophylaxis, maintaining normal body temperature, and controlling blood sugar—remains essential [7,67].

These findings further emphasize the need for close and continuous monitoring of surgical site infections and antimicrobial resistance patterns. Our results resonate with reports by Rubulotta et al., who highlighted the role of innovative point-of-care diagnostics (SPOCD) in monitoring infection rates during the COVID-19 pandemic [68]. Their work demonstrated that, even in low-resource settings, the judicious use of diagnostic technology can sustain effective microbiological surveillance and stewardship, enabling timely clinical decision-making and improved infection control strategies.

In this context, reinforcing perioperative antimicrobial stewardship, targeted prophylaxis, antiseptic wound care strategies, and microbiological monitoring remain essential to mitigate a post-pandemic resurgence in SSI. The persistence of resistant Gram-negative pathogens such as *P. aeruginosa* underscores the importance of sustained surveillance and adaptive stewardship strategies beyond pandemic periods [55].

Future multicenter studies are necessary to validate these findings, connect SSI trends to surgical activity, and assess the durability of post-pandemic reductions in resistance.

## 5. Conclusions

This six-year retrospective analysis demonstrated that the COVID-19 pandemic significantly impacted the dynamics of SSIs in patients undergoing conventional surgeries.

During the pandemic period (2020–2022), the number of SSIs increased, followed by a decline afterward, reflecting the disruption and subsequent recovery of surgical services. Notably, patients during the pandemic were younger. They had shorter hospital stays, which may be associated with changes in healthcare-seeking behavior and the implementation of stricter inpatient management policies, although demographic and comorbidity profiles remained stable.

The most prevalent pathogens were *Pseudomonas aeruginosa* and *Enterococcus* spp., which together accounted for nearly half of all infections throughout the study periods. Their predominance, particularly in cases of chronic limb-threatening ischemia and soft-tissue infections, highlights the persistence of opportunistic and environment-adapted bacteria in surgical wounds.

Importantly, there was a statistically significant decline in fluoroquinolone resistance among *Enterococcus* spp. post-pandemic, dropping from over 50% to less than 10% (*p* = 0.030). This observation coincided with the post-pandemic period and may reflect changes in antimicrobial prescribing practices; however, a causal relationship cannot be established in this observational study.

Although the frequency of emergency surgeries and the severity of cases increased during the pandemic, postoperative ICU admissions and mortality rates remained low, indicating that overall surgical outcomes were maintained despite operational challenges.

In summary, the COVID-19 pandemic reshaped the epidemiology of SSIs by altering the mix of surgical cases, bacterial ecology, and patterns of antimicrobial resistance. These findings underscore the need for continuous microbiological surveillance, adaptable infection control measures, and reinforced antibiotic stewardship to sustain the observed improvements in resistance profiles after the pandemic. Future multicenter, prospective studies are needed to validate these trends and to elucidate better the factors underlying changes in resistance patterns in surgical settings.

## Figures and Tables

**Table 1:** Demographic and Clinical Characteristics.

Variables	Pre-Pandemic *n* = 72	Pandemic *n* = 48	Post-Pandemic *n* = 53	*p*
Age (years, M ± SD)	66.92 ± 10.36	62.29 ± 14.72	64.29 ± 11.66	0.117
Gender				
Male	31 (43.1%)	29 (60.4%)	24 (45.3%)	0.149
Female	41 (56.9%)	19 (39.6%)	29 (54.7%)	
Environment				
Urban	41 (56.9%)	26 (54.2%)	31 (58.5%)	0.907
Rural	31 (43.1%)	22 (45.8%)	22 (41.5%)	
Charlson Index > 3	37 (51.4%)	19 (39.6%)	22 (41.5%)	0.4

Legend: M ± SD = mean ± standard deviation. Percentages are calculated based on the total number of patients in each period (Pre-Pandemic *n* = 72, Pandemic *n* = 48, Post-Pandemic *n* = 53).

**Table 2:** Diagnostic Categories.

Diagnostics	Pre-Pandemic *n* = 72	Pandemic *n* = 48	Post-Pandemic *n* = 53	*p*
Abdominopelvic neoplasm	14 (19.4%)	4 (8.3%)	10 (18.9%)	0.012
Acute abdomen	2 (2.8%)	5 (10.4%)	4 (7.5%)	
Abdominal wall defect	8 (11.1%)	4 (8.3%)	6 (11.3%)	
Chronic limb-threatening ischemia	36 (50%)	14 (29.2%)	15 (28.3%)	
Soft tissue infections	3 (4.2%)	9 (18.8%)	11 (20.8%)	
Venous leg ulcer	7 (9.7%)	6 (12.5%)	6 (11.3%)	
Others	2 (2.8%)	6 (12.5%)	1 (1.9%)	

Data are presented as number (percentage). Percentages are calculated based on the total number of patients in each period (Pre-Pandemic *n* = 72, Pandemic *n* = 48, Post-Pandemic *n* = 53). The *p* value represents overall differences across the three study periods (Pre-Pandemic, Pandemic, and Post-Pandemic), calculated using the Chi-square test for categorical variables.

**Table 3:** Bacterial species distribution among the three studied timeframes.

Bacterial Species	Pre-Pandemic	Pandemic	Post-Pandemic	*p*
*Pseudomonas aeruginosa*	19 (26.4%)	7 (14.6%)	13 (24.5%)	0.291
*Enterococcus* spp.	12 (16.7%)	15 (31.3%)	11 (20.8%)	0.162
*Escherichia coli*	14 (19.4%)	12 (25%)	6 (11.3%)	0.202
*Klebsiella* spp.	9 (12.5%)	6 (12.5%)	11 (20.8%)	0.375
*Staphylococcus aureus*	16 (22.2%)	6 (12.5%)	13 (24.5%)	0.278
*Proteus mirabilis*	8 (11.1%)	2 (4.2%)	5 (9.4%)	0.405
*Acinetobacter baumannii*	7 (9.7%)	1 (2.1%)	3 (5.7%)	0.263
*Morganella morganii*	1 (1.4%)	4 (8.3%)	4 (7.5%)	0.160

Data are presented as numbers (percentages). Percentages are calculated based on the total number of bacterial isolates identified in each period, including both monomicrobial and polymicrobial infections. As a result, percentages may exceed 100% because multiple bacterial species can be isolated from the same patient.

**Table 4:** *Pseudomonas aeruginosa* incidence over the studied periods.

*Pseudomonas aeruginosa* Postoperative Wound Infection	Pre-Pandemic *n* = 19	Pandemic *n* = 7	Post-Pandemic *n* = 13	*p*
Abdominopelvic neoplasm	4 (21.1%)	0	0	0.007
Acute abdomen	1 (5.3%)	0	2 (15.4%)	
Abdominal wall defect	0	0	3 (23.1%)	
Chronic limb-threatening ischemia	7 (36.8%)	2 (28.6%)	0	
Soft tissue infections	0	3 (42.9%)	2 (15.4%)	
Venous leg ulcer	7 (36.8%)	2 (28.6%)	6 (46.2%)	

Data are presented as numbers (percentages). Percentages are calculated based on the total number of postoperative wound infections caused by *Pseudomonas aeruginosa* in each study period (Pre-Pandemic *n* = 19, Pandemic *n* = 7, Post-Pandemic *n* = 13). The *p* value represents overall differences across the three study periods (Pre-Pandemic, Pandemic, and Post-Pandemic), calculated using the Chi-square test for categorical variables.

**Table 5:** *Enterococcus* spp. incidence over the studied periods.

*Enterococcus* spp. Postoperative Wound Infection	Pre-Pandemic *n* = 12	Pandemic *n* = 15	Post-Pandemic *n* = 11	*p*
Abdominopelvic neoplasm	4 (33.3%)	2 (13.3%)	4 (36.4%)	0.178
Acute abdomen	0	3 (20%)	1 (9.1%)	
Abdominal wall defect	1 (8.3%)	1 (6.7%)	0	
Chronic limb-threatening ischemia	7 (58.3%)	3 (20%)	5 (45.5%)	
Soft tissue infections	0	4 (26.7%)	0	
Venous leg ulcer	0	1 (6.7%)	1 (9.1%)	
Others	0	1 (6.7%)	0	

Data are presented as numbers (percentages). Percentages are calculated based on the total number of postoperative wound infections caused by *Enterococcus* spp. in each study period (Pre-Pandemic n = 12, Pandemic n = 15, Post-Pandemic n = 11). The *p* value represents overall differences across the three study periods (Pre-Pandemic, Pandemic, and Post-Pandemic), calculated using the Chi-square test for categorical variables.

**Table 6:** *Pseudomonas aeruginosa* antibiotic resistance profile.

	Pre-Pandemic *n* = 19	Pandemic *n* = 7	Post-Pandemic *n* = 13	*p*
Amikacin	19 (100.0%)	7 (100.0%)	8 (61.5%)	0.689
Cefepime	17 (89.5%)	4 (57.1%)	7 (53.8%)	
Ceftazidime	14 (73.7%)	4 (57.1%)	5 (38.5%)	
Ciprofloxacin	10 (52.6%)	4 (57.1%)	5 (38.5%)	
Colistin	11 (57.9%)	2 (28.6%)	4 (30.8%)	
Gentamicin	14 (73.7%)	4 (57.1%)	9 (69.2%)	
Imipenem	11 (57.9%)	3 (42.9%)	6 (46.2%)	
Levofloxacin	8 (42.1%)	2 (28.6%)	1 (7.7%)	
Meropenem	16 (84.2%)	4 (57.1%)	7 (53.8%)	
Piperacillin–Tazobactam	15 (78.9%)	4 (57.1%)	6 (46.2%)	
Piperacillin	16 (84.2%)	3 (42.9%)	5 (38.5%)	

Percentages represent antimicrobial susceptibility rates and refer to the proportion of tested isolates that were susceptible to each antibiotic during each study period.

**Table 7:** *Enterococcus* spp. antibiotic resistance profile.

Antibiotic	Pre-Pandemic *n* = 12	Pandemic *n* = 15	Post-Pandemic *n* = 11	*p*
Ampicillin	5 (41.7%)	11 (78.6%)	9 (81.8%)	0.672
Ciprofloxacin	0 (0.0%)	2 (14.3%)	4 (36.4%)	
Gentamicin	8 (66.7%)	10 (71.4%)	9 (81.8%)	
Levofloxacin	2 (16.7%)	4 (28.6%)	5 (45.5%)	
Linezolid	10 (83.3%)	12 (85.7%)	10 (90.9%)	
Tigecycline	7 (58.3%)	14 (100.0%)	8 (72.7%)	
Vancomycin	10 (83.3%)	13 (92.9%)	11 (100.0%)	

Percentages represent the proportion of *Enterococcus* spp. isolates resistant to each antimicrobial agent among the isolates tested in each study period.

**Table 8:** *Pseudomonas aeruginosa* resistance profile.

Antibiotic	Pre-Pandemic *n* = 19	Pandemic *n* = 7	Post-Pandemic *n* = 13	*p*
Cefepime	1 (5.3%)	2 (28.6%)	1 (7.7%)	
Ceftazidime	1 (5.3%)	2 (28.6%)	1 (7.7%)	
Imipenem	3 (15.8%)	2 (28.6%)	2 (15.4%)	
Meropenem	2 (10.5%)	2 (28.6%)	1 (7.7%)	
Ticarcillin/ Clavulanic Acid	1 (5.3%)	–	2 (15.4%)	0.421
Piperacillin	3 (15.8%)	3 (42.9%)	3 (23.1%)	
Piperacillin/ Tazobactam	1 (5.3%)	1 (14.3%)	1 (7.7%)	
Tobramycin	1 (5.3%)	–	–	
Ciprofloxacin	4 (21.1%)	2 (28.6%)	4 (30.8%)	
Levofloxacin	6 (31.6%)	2 (28.6%)	–	
Norfloxacin	1 (5.3%)	–	–	
Colistin	–	–	1 (7.7%)	

Percentages represent the proportion of *Pseudomonas aeruginosa* isolates resistant to each antimicrobial agent among the isolates tested in each study period.

**Table 9:** *Pseudomonas aeruginosa* intermediate resistance profile.

Antibiotic	Pre-Pandemic *n* = 19	Pandemic *n* = 7	Post-Pandemic *n* = 13	*p*
Imipenem	4 (21.1%)	2 (28.6%)	3 (23.1%)	
Meropenem	1 (5.3%)	1 (14.3%)	–	
Cefepime	–	1 (14.3%)	3 (23.1%)	
Ceftazidime	1 (5.3%)	1 (14.3%)	4 (30.8%)	
Piperacillin/ Tazobactam	2 (10.5%)	2 (28.6%)	3 (23.1%)	0.712
Piperacillin	–	1 (14.3%)	2 (15.4%)	
Ciprofloxacin	1 (5.3%)	1 (14.3%)	3 (23.1%)	
Levofloxacin	1 (5.3%)	1 (14.3%)	2 (15.4%)	
Gentamicin	1 (5.3%)	–	–	

Data are presented as numbers (percentages). Percentages represent the proportion of *Pseudomonas aeruginosa* intermediate resistance to each antimicrobial agent among the isolates tested in each study period.

**Table 10:** *Enterococcus* spp. resistance profile over the three timeframes.

Antibiotic	Pre-Pandemic *n* = 12	Pandemic *n* = 15	Post-Pandemic *n* = 11
Ampicillin	4 (33.3%)	2 (13.3%)	1 (9.1%)
Ciprofloxacin	6 (50.0%)	8 (53.3%)	1 (9.1%)
Gentamicin	2 (16.7%)	4 (26.7%)	2 (18.2%)
Imipenem	–	–	1 (9.1%)
Levofloxacin	6 (50.0%)	8 (53.3%)	1 (9.1%)
Tigecycline	–	–	2 (18.2%)

Data are presented as numbers (percentages). Percentages represent the proportion of *Enterococcus* spp. resistance to each antimicrobial agent among the isolates tested in each study period.

**Table 11:** Antibiotic intermediate sensitivity.

Antibiotic	Pre-Pandemic *n* = 12	Pandemic *n* = 15	Post-Pandemic *n* = 11
Ampicillin	2 (16.7%)	–	–
Ciprofloxacin	3 (25.0%)	4 (26.7%)	–
Gentamicin	1 (8.3%)	–	–
Imipenem	–	–	1 (9.1%)
Levofloxacin	3 (25.0%)	2 (13.3%)	–

Data are presented as numbers (percentages). Percentages represent the proportion of *Enterococcus* spp. intermediate sensitivity to each antimicrobial agent among the isolates tested in each study period.

## Data Availability

The datasets generated and/or analyzed during this study are available from the first author upon reasonable request.

## Abbreviations

CCI	Charlson Comorbidity Index
CDC	The Centers for Disease Control and Prevention
CLSI	Clinical and Laboratory Standards Institute
CI	Confidence intervals
EMR	Electronic Medical Record
ERAS	Enhanced Recovery After Surgery
EU GDPR	European Union General Data Protection Regulation
HAI	Hospital-Acquired Infections
IBM SPSS	IBM Statistical Package for the Social Sciences
ICU	Intensive Care Unit
M ± SD	Mean ± Standard Deviation
RT-PCR	Reverse transcription polymerase chain reaction
SSIs	Surgical site infections
WHO	World Health Organisation
